# Theatre-Based Carbon Footprint of Five Common Orthopaedic Trauma Procedures: A Single-Centre Comparative Analysis

**DOI:** 10.7759/cureus.114801

**Published:** 2026-08-19

**Authors:** Charles Gamble, Pushkar Joshi, Preetham Kodumuri

**Affiliations:** 1 Trauma and Orthopaedics, Wrexham Maelor Hospital, Wrexham, GBR; 2 Trauma and Orthopaedics, Wrightington Hospital, Wigan, GBR

**Keywords:** carbon footprint, greenhouse gas emissions, operating theatre, orthopaedic trauma, sustainability

## Abstract

Introduction* *

Operating theatres are resource-intensive environments and contribute disproportionately to healthcare-related carbon emissions. While the environmental impact of elective orthopaedic surgery has been described, there are limited procedure-level data for orthopaedic trauma surgery.

Methods

A single-centre, retrospective, theatre-based carbon footprint analysis was undertaken for five common orthopaedic trauma procedures performed in 2023: hip hemiarthroplasty, sliding hip screw fixation, femoral intramedullary nailing, ankle fracture fixation, and minor trauma procedures. Consumables and selected instrument-related items were weighed and assigned material-specific emission factors. Theatre energy and water use were estimated using assumed theatre-level averages and allocated by operative duration. Emissions were expressed as kilograms of carbon dioxide equivalent (kgCO₂e) per procedure.

Results

Theatre-based emissions ranged from 33.3 to 49.8 kgCO₂e per procedure. Hip hemiarthroplasty had the highest emissions, while minor procedures had the lowest. Consumables accounted for 34% of total emissions, with single-use gowns and drapes contributing 37% of consumable-related emissions. Allocated theatre energy and water use accounted for 55.8% of emissions and were similar across procedures.

Conclusion

Common orthopaedic trauma procedures have similar theatre-based carbon footprints, with variation driven primarily by consumable use. Although absolute emissions are underestimated owing to exclusion of supply-chain sources, this analysis identifies intra-operative emission hotspots that may inform local quality improvement initiatives.

## Introduction

Healthcare systems are substantial contributors to global greenhouse gas emissions, with the National Health Service (NHS) being the largest public-sector source in the United Kingdom [1,2]. Operating theatres are particularly resource-intensive, owing to high ventilation requirements, continuous lighting, sterilisation processes, and extensive use of single-use consumables [3,4].

Trauma and orthopaedic surgery constitutes a major component of NHS operative activity, particularly through high-volume emergency pathways, such as hip fracture care [5]. Despite increasing interest in sustainable surgical practice, most procedure-level carbon footprint analyses in orthopaedics have focused on elective surgery [6-8]. There remains a relative paucity of data relating specifically to orthopaedic trauma procedures, which differ from elective practice in urgency, case mix, and opportunities for preoperative optimisation.

Life cycle assessment frameworks provide a structured approach to quantifying carbon emissions but are challenging to apply comprehensively at the level of individual surgical procedures [9]. Supply-chain emissions related to manufacture, transport, and disposal typically account for the majority of healthcare carbon footprints but are rarely available at sufficient resolution to support full pathway analyses [1,10]. Consequently, theatre-based analyses are commonly used to identify comparative intra-operative emission sources while acknowledging that absolute emissions will be underestimated [11].

The aim of this study was to quantify and compare theatre-based greenhouse gas emissions associated with five commonly performed orthopaedic trauma procedures, identify principal intra-operative contributors, and provide comparative data to inform future quality improvement and research.

This article was previously presented as an oral poster at the Association of Surgeons in Training (ASiT) Annual Conference on 7 March 2026.

## Materials and methods

A single-centre, retrospective, theatre-based carbon footprint analysis was conducted for orthopaedic trauma procedures performed between January and December 2023 at a UK district general hospital.

Five procedure groups were included: hip hemiarthroplasty, sliding hip screw fixation, femoral intramedullary nailing, ankle fracture fixation, and minor orthopaedic trauma procedures (including Kirschner wire fixation, wound washout, and implant removal).

The system boundary was restricted to activities occurring within the operating theatre, from patient entry to transfer to recovery. Included emission sources were consumables opened and used during the procedure, selected surgical instrument-related items, anaesthetic-related disposables and medication packaging, and allocated theatre energy and water use. Supply-chain emissions, including manufacture and transport of products, off-site laundering, waste management, patient and staff travel, and capital infrastructure, were not quantified [1,10].

For each procedure group, 10 representative cases were sampled to estimate typical consumable use and operative duration. Items opened and used were weighed and categorised by material type, with all items weighed to the nearest 1 g. Item-level emissions were calculated using published material-specific emission factors derived from the Inventory of Carbon and Energy and related datasets [7,11]. Emission factors were multiplied by the weight of each item and aggregated to determine the total kilograms of carbon dioxide equivalent (kgCO₂e) per procedure. 

Theatre electricity, heating, ventilation, lighting, and water use were estimated using assumed theatre-level averages and allocated proportionally by operative duration. Sterilisation-related emissions were allocated per case according to the number of instrument trays opened. Volatile anaesthetic gases and nitrous oxide were not included owing to lack of reliable case-level consumption data [4].

## Results

The theatre-based carbon footprint varied across the five orthopaedic trauma procedures analysed, ranging from 33.3 to 49.8 kgCO₂e per procedure. Ten cases per procedure were analysed (total n = 50). Hip hemiarthroplasty was associated with the highest emissions (49.8 kgCO₂e), while minor orthopaedic trauma procedures had the lowest (33.3 kgCO₂e). Sliding hip screw fixation, femoral intramedullary nailing, and ankle fracture fixation demonstrated intermediate values (Table 1).

**Table 1:** Theatre-based carbon emissions for orthopaedic trauma procedures Theatre-based emissions per procedure in kilograms of carbon dioxide equivalent (kgCO₂e). Energy and water values are allocated estimates based on assumed theatre-level averages. Supply-chain emissions and volatile anaesthetic gases were not included.

	Procedure type				
	Hemiarthroplasty	Sliding hip screw fixation	Intramedullary nailing	Ankle fracture fixation	Minor procedure
Anaesthetic (kgCO_2_e)	3.973	3.973	3.973	3.667	3.667
Surgical instruments (kgCO_2_e)	0.09	0.049	0.053	0.051	0.049
Consumables (kgCO_2_e)	21.786	12.071	15.547	11.879	9.775
Lighting (kgCO_2_e)	4.594	4.594	4.594	4.594	4.594
Ventilation (kgCO_2_e)	3.031	3.031	3.031	3.031	0.725
Heating (kgCO_2_e)	5.595	5.595	5.595	5.595	5.595
Water (kgCO_2_e)	8.493	8.493	8.493	8.493	8.493
Sterilisation (kgCO_2_e)	2.218	0.918	0.918	0.918	0.437
Total (kgCO_2_e)	49.78	38.724	42.204	38.228	33.335

Across all procedures, allocated theatre energy and water use accounted for 55.8% of total emissions, followed by consumables at 34.0%. Surgical instrument-related items contributed less than 1% of total emissions (Table 1).

Consumables represented the principal source of variation between procedures. Hip hemiarthroplasty demonstrated the highest consumable-related emissions (21.8 kgCO₂e), compared with 9.8 kgCO₂e for minor procedures (Table 1). Across all procedures, single-use gowns and drapes accounted for 37% of consumable-related emissions (mean 4.43 kgCO₂e per procedure). Packaging materials contributed 4.4% on average (0.53 kgCO₂e).

Within allocated theatre energy and water emissions, water use accounted for 38.2%, heating 25.1%, lighting 20.6%, ventilation 11.3%, and sterilisation 4.7%. Sterilisation-related emissions varied by the number of instrument trays used, ranging from 0.44 kgCO₂e for minor procedures to 2.22 kgCO₂e for hip hemiarthroplasty (Table 1).

## Discussion

This study demonstrates that five common orthopaedic trauma procedures have similar theatre-based carbon footprints, with emissions ranging from approximately 33 to 50 kgCO₂e per procedure. Consumables and allocated theatre energy and water use were the dominant contributors, with variation between procedures driven primarily by consumable use rather than differences in theatre infrastructure.

These findings are consistent with previous surgical and orthopaedic carbon footprint analyses, which have identified consumables and theatre energy use as major contributors to peri-operative emissions [6-8,12]. The present analysis extends this literature to orthopaedic trauma surgery, an area of high clinical volume that has received comparatively limited attention.

National analyses of the NHS carbon footprint demonstrate that supply-chain (Scope 3) emissions account for the majority of healthcare-related emissions [1,10]. Against this backdrop, the theatre-based emissions quantified here represent only a partial view of the total carbon footprint associated with orthopaedic trauma procedures. We deliberately avoided modelling procedure-level supply-chain emissions to prevent false precision and because national data cannot be reliably disaggregated to individual procedures. This approach is consistent with prior surgical sustainability work employing partial system boundaries [11].

The finding that consumables drive variation between procedures suggests a pragmatic focus for local quality improvement. Evidence supports potential environmental benefits of reusable textiles compared with disposable options, although such changes require careful consideration of infection control and local processing pathways [12-14]. Similarly, optimising instrument trays has been associated with improved theatre efficiency and cost reductions, with potential sustainability benefits [15-17].

This study has several limitations, including its single-centre design, reliance on assumed theatre-level energy and water estimates rather than direct measurement, exclusion of volatile anaesthetic gases, and omission of supply-chain emissions. No formal uncertainty analysis or health economic evaluation was performed. Future research, whilst addressing these limitations, would be further strengthened by a multi-centre approach to improve the generalisability and applicability across different healthcare systems. 

## Conclusions

In conclusion, common orthopaedic trauma procedures have similar theatre-based carbon footprints, with consumables and allocated theatre energy and water use as the dominant contributors. Although absolute emissions are underestimated, this analysis identifies intra-operative emission hotspots and provides a foundation for future multi-centre studies incorporating direct metering, anaesthetic gas data, and full care-pathway analyses. These findings underscore the importance of a nuanced approach to sustainability in trauma care, recognising that different procedures may require tailored strategies for optimal resource management and emissions reduction. The successful implementation of sustainability practices in trauma and orthopaedics will require a collaborative approach amongst healthcare professionals, hospital administrators, and sustainability experts to ensure environmental considerations are integrated into clinical decision-making without compromising patient outcomes.
